# Photocatalytic properties of BiFeO_3_ powders synthesized by the mixture of CTAB and Glycine

**DOI:** 10.1038/s41598-023-39622-4

**Published:** 2023-07-31

**Authors:** N. Asefi, M. Hasheminiasari, S. M. Masoudpanah

**Affiliations:** School of Metallurgy and Materials Engineering, Iran University of Science and Technology (IUST), Narmak, Tehran, Iran

**Keywords:** Photocatalysis, Synthesis and processing, Nanoscale materials

## Abstract

Highly pure BiFeO_3_ (BFO) powders were prepared by the solution combustion synthesis method using cetyltrimethylammonium bromide (CTAB) and glycine as fuels at various fuel-to-oxidant (φ) ratios. Microstructural characteristics, morphology, optical properties, and thermal analysis were studied by X-ray diffraction (XRD), scanning electron microscopy (SEM), diffuse reflectance spectroscopy (DRS), and differential thermal/thermogravimetry (DTA/TGA), respectively. The combusted powders prepared at different fuel content contained a small amount of impurity phases such as Bi_24_Fe_2_O_39_ and Bi_2_Fe_4_O_9_. During the calcination of BFO powders at 600 °C for 1 h, a nearly pure BFO phase was produced. Combusted powders photodegraded about 80% of methylene blue dye at φ = 2 through 90 min of visible light irradiation.

## Introduction

Single-phase BiFeO_3_ (BFO) is a multiferroic material with distorted rhombohedral and perovskite structures exhibiting the R3c space group. Due to its ferroelectric performance at high Curie temperatures up to 830 °C and antiferromagnetic behavior under its Neel temperature of 370 °C, this material is considered for non-volatile memory devices, photovoltaics, sensors, and spintronics^1–4^. It is also known that these abundant and interesting compounds with the perovskite structure exhibit improved compositional and structural tunability^5,6^. Because of its narrow bandgap in the range of 2.2–2.8 eV and high chemical stability, BFO has been considered a visible light photocatalyst to degrade organic pollutants^7^. Many photocatalysts, such as TiO_2_, ZnO, CdS, ZnS, etc., have been used to photodegrade dyes under ultraviolet (UV) light irradiation^8–13^. However, UV only spans a small portion (~ 4%) of the sunlight spectrum; thus, many efforts have been made to develop visible-light catalysts covering a broader range^14–19^.

Impurity phases such as Bi_2_O_3_, Bi_2_Fe_4_O_9_, and Bi_24_Fe_2_O_39_ appear during the synthesis of BFO due to their phase formation kinetics. Therefore, many researchers developed various synthesis routes to remove these secondary phases. Hydrothermal methods^20,21^, polymer assisted hydrothermal^22^, sol–gel^23^, co-precipitation^24–26^, aerosol-spraying, electrospinning^27^, solvothermal route^28^, and solution combustion^29^ were used to synthesize pure BFO.

Developing simple, environmentally safe, and energy-efficient methods to synthesize a pure BFO powder is of great interest. Solution combustion synthesis (SCS) is a simple, relatively cheap, and fast chemical process to produce various nanomaterials^30^. A self-propagating exothermic reaction occurs between the mixture of metal nitrates and different organic fuels (e.g., glycine, citric acid, urea, etc.), releasing an enormous amount of gaseous products^29^.

Among different organic fuels, glycine is an amino acid that facilitates the formation of a metal ion complex in the solution owing to its carboxylic acid and amino groups at opposite ends of the molecule^31^. Likewise, Cetyltrimethylammonium bromide (CTAB) is a cationic surfactant with a high decomposition temperature that is extensively used to control particle shapes, size, and microstructure by minimizing the precursor’s surface tension^32^. BFO has been synthesized by glycine fuel through microwave-assisted solution combustion with some impurity phases such as Bi_2_Fe_4_O_9_ and Bi_24_Fe_2_O_39_^33^. In our previous works, BFO was synthesized using various single and mixed fuels at a constant fuel to oxidant ratio of 1, but in this work different fuel-to-oxidant ratios (φ) were varied from 0.5 to 2^32–34^.

Nevertheless, combining different fuels might be more effective than individual fuels via improved control over the reaction temperature, the type, and the amount of gaseous products released. Therefore, in this work, glycine and CTAB were mixed at various fuel-to-oxidant amounts in the uni-molar ratio to synthesize nearly pure and single-phase BFO.

## Experimental procedure

### Synthesis route

Analytical grades Fe(NO3)3.9H2O, Bi(NO3)2.5H2O, CTAB [(C16H33)N(CH3)3]Br (> 99%), glycine (C2H5NO2), were purchased from Merk Co. without any further purification. Whereby HNO3 (68 wt %) was added to dissolve bismuth nitrate. The required amount of Bi(NO3)0.5H2O and Fe(NO3)0.9H2O, cetyltrimethylammonium bromide ([(C16H33)N(CH3)3]Br), and glycine (C2H5NO2) were prepared by dissolving 15 mL of 3 mol L^−1^ of HNO3 in various fuel-to-oxidant ratios of (φ = 0.5, 0.75, 1 and 2). H_2_O, CO_2_, Br_2_, and N_2_ are assumed to be the gaseous products of the combustion reaction, where the type of gaseous products and adiabatic temperature are controlled by the fuel-to-oxidant ratio (φ).

$$\begin{aligned} & {\text{Bi}}\left( {{\text{NO}}_{{3}} } \right)_{{3}} + {\text{ Fe}}\left( {{\text{NO}}_{{3}} } \right)_{{3}} + \frac{1}{2}\left( {\frac{15}{{59}}\varphi {\text{ C}}_{{{19}}} {\text{H}}_{{{36}}} {\text{NBr}} + \frac{10}{3}\varphi {\text{ C}}_{{2}} {\text{H}}_{{5}} {\text{NO}}_{{2}} } \right) + \frac{15}{2} \left( {\varphi - {1}} \right){\text{ O}}_{{2}} \to {\text{BiFeO}}_{{3}} \\ & \quad + \frac{2035}{{354}}\varphi {\text{CO}}_{{2}} + { }\frac{2285}{{354}}\varphi {\text{H}}_{{2}} {\text{O}} + \, \left( {{3} + \frac{635}{{708}}{ }\varphi } \right){\text{N}}_{{2}} + \frac{15}{{236}}{ }\varphi {\text{ Br}}_{{2}} \\ \end{aligned}$$

8500S SHIMADZU spectrophotometer recorded IR spectra in the range of (400–4000 cm^−1^). Differential thermal (DTA) and thermogravimetry analysis (TGA) were used to study the combustion behavior in the air with a heating rate of 5 ˚C/min on an STA Ba¨HR 503 instrument. Microstructure and phase evolution were examined by X-ray diffraction (PANalytical X’pert, CuKa = 1.54060 A°). Crystallite sizes were also calculated using the raw data from XRD using the Williamson-Hall method. Field emission scanning electron microscopy (FESEM) was acquired to characterize the morphology of the as-combusted powders by TESCAN Vega II. UV–Vis diffuse reflectance spectrum (DRS) was acquired to measure the bandgap and visible light absorption of the powders by a Shimadzu UV–Vis 52550 spectrophotometer in the wavelength range of (300–800 nm). A Tauc’s plot is used to determine the optical bandgap of semiconductors. Typically, a Tauc’s plot shows the quantity hν (the photon energy) on the (x-coordinate) and the quantity (αhν)^1/2^ on the (y-coordinate), where α is the absorption coefficient of the material. Thus, extrapolating this linear region to the abscissa yields the energy of the optical bandgap of the semiconductor material.

### Photocatalytic performance

In the presence of combusted BFO powders, the methylene blue (MB) dye was broken down by visible light irradiation from two 100 W Xenon lamps with a cutoff ultraviolet filter.100 mg of BFO catalyst was dispersed in 100 mL of methylene blue (15 mg/L) in the company of 0.1 mL of H_2_O_2_ (30%) and stirred for 60 min in the dark to obtain the adsorption/desorption equilibrium. Furthermore, the pH of the solution was adjusted by HCl (37 wt%). BFO powders were separated by centrifugation at 6000 rpm for 10 min, followed by MB concentration monitoring on a PG Instruments Ltd. T80-UV/Vis spectrophotometer.

## Results and discussion:

**Figure 1:**
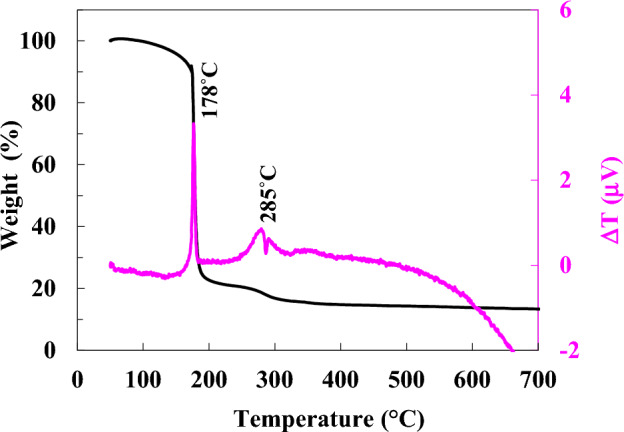
TGA/DTA curves of the dried gel prepared by a mixture of CTAB and glycine fuels at φ = 1.

Figure 1 illustrates the thermal analysis of dried gel produced by a mixture of glycine and CTAB fuels at φ = 1. A slight drop (~ 9%) in the gel’s mass is possibly due to the evaporation of absorbed water. A sharp decline at about 178 °C, possibly triggered by the exothermic reaction between metal nitrates and glycine CTAB fuels. This enormous drop (~ 70%) in the gel mass is because of a combustion reaction that released a large amount of gaseous products such as CO_2_, H_2_O, N_2_, Br_2_, etc.

Based on previous findings^34–36^, glycine has a lower decomposition temperature compared to CTAB, with a fast combustion reaction rate showing sharp weight loss in the gel. Similarly, when the mixture of glycine and CTAB is used as a fuel, colossal weight loss seems to be dominated by the presence of glycine rather than CTAB. Furthermore, a smaller exothermic peak at 285 °C with a gradual weight loss might be attributed to the slow oxidation reaction of residual organics that remained in the gel^37^.

**Figure 2:**
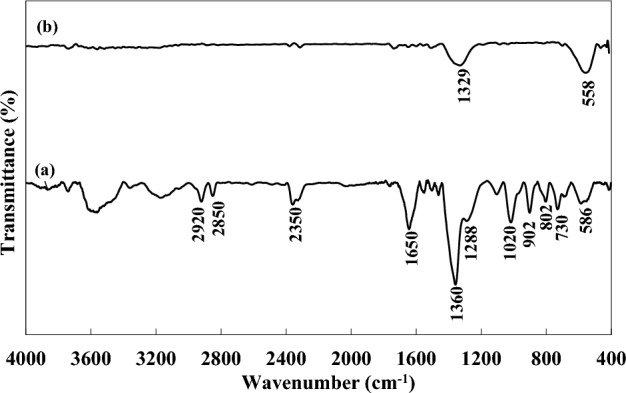
FTIR spectra of (**a**) dried gel and (**b**) the as-combusted BFO powders at φ = 1.

The FTIR spectra of dried gel and as-combusted powders prepared by the glycine and CTAB fuel mixture at φ = 1 are illustrated in Fig. 2. The broad vibrational stretching modes in the range of 3200–3700 cm^−1^ correspond to the absorption of hydroxyl groups of water molecules that are omitted in the combusted BFO powders^38^. The stretching vibration of C-H bonds in CTAB molecules can lead to the formation of bands at 2920 and 2850 cm^−1^^39^. The vibrational band at 1350 cm^−1^ is due to the attachment of CO_3_^2−^ groups to the cations^38^. The adsorption bands at 1650 cm^−1^, 1360 cm^−1^, 902 cm^−1^, 802 cm^−1^, and 730 cm^−1^ confirm the formation of NO_3_^−^ connected to the CTAB and glycine molecules in the dried gel^40^. Stretching bands at 1757 cm^−1^ and 1556 cm^−1^ resemble the existence of COO^−^ groups formed through the oxidation of CTAB molecules^41^. Peaks at 1105 cm^−1^ and 1020 cm^−1^ confirm the presence of NH_2_ groups. Carboxylate groups can chelate cations, leading to the absorption band at 586 cm^−1^, which corresponds to metal–oxygen bonds^42^. Strong peaks at 557 cm^−1^ and 465 cm^−1^ of combusted powder can be assigned to the vibrational bending and stretching of Fe–O in the octahedral FeO6 groups in the perovskite structure^43^.

**Figure 3:**
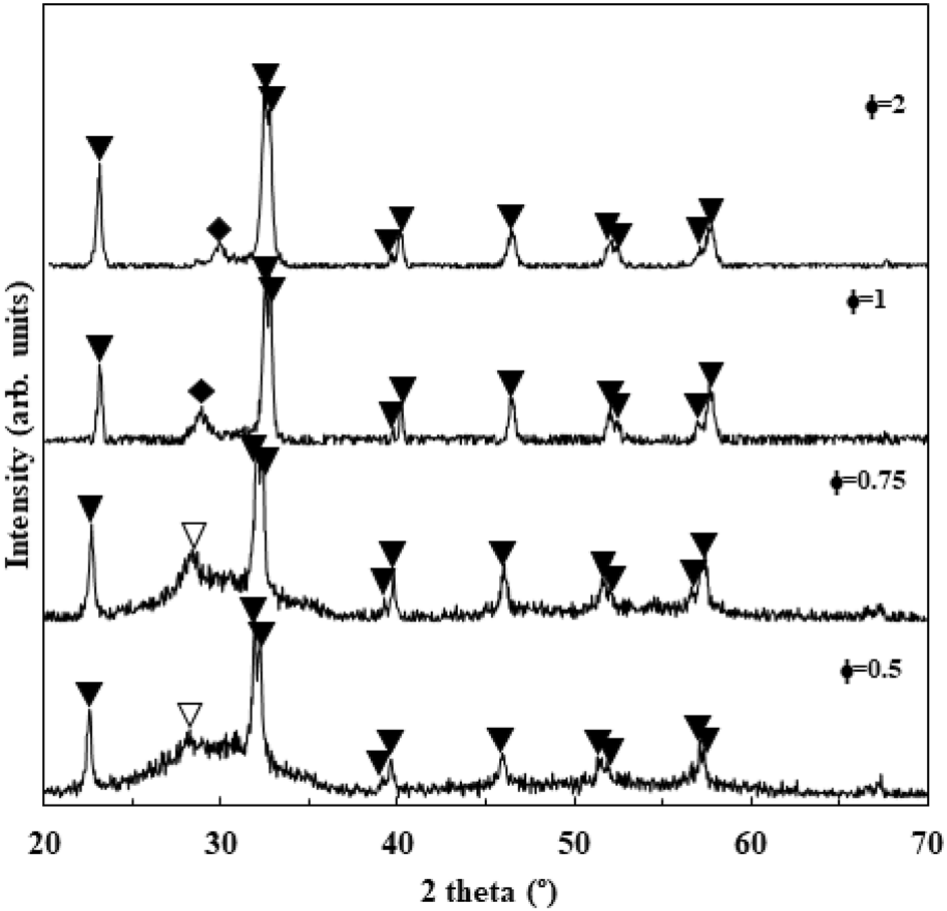
XRD patterns of the as-combusted BFO powders using glycine and CTAB fuel content. (filled down pointing triangle: BiFeO_3_, open down pointing triangle: Bi_2_Fe_4_O_9_, filled diamond: Bi_24_Fe_2_O_39_).

XRD patterns of conventionally combusted BFO powders at the various φ values are depicted in Fig. 3. The combusted powders at φ = 0.5 and φ = 0.75 are semi-amorphous due to their incomplete combustion reaction and low adiabatic combustion temperature. Bi_2_Fe_4_O_9_ (JCDPS Card No. 00-020-0836) impurity phase is presented at φ values of 0.5 and 0.75. However, maximum adiabatic temperature occurs at φ = 1, leading to a well crystalline pattern. Bi_24_Fe_2_O_39_ (JCDPS Card No. 00-042-0201) was the only impurity phase formed at the φ values of 1 and 2.

Bi_2_O_3_ and Fe_2_O_3_, as the transitional phases, can take part in the solid reaction of (Bi_2_O_3_ + Fe_2_O_3_ → 2 BiFeO_3_) to produce the BiFeO_3_ phase. Nevertheless, the formation of impurity phases such as Bi_2_Fe_4_O_9_ and Bi_24_Fe_2_O_39_ can be ascribed to the insufficiency of Bi_2_O_3_ and Fe_2_O_3_ phases initiated by the phase segregation^44^:

$${\text{Bi}}_{{2}} {\text{O}}_{{3}} + {\text{ 2 Fe}}_{{2}} {\text{O}}_{{3}} \to {\text{ Bi}}_{{2}} {\text{Fe}}_{{4}} {\text{O}}_{{9}}$$

$${\text{12 Bi}}_{{2}} {\text{O}}_{{3}} + {\text{ Fe}}_{{2}} {\text{O}}_{{3}} \to {\text{ Bi}}_{{{24}}} {\text{Fe}}_{{2}} {\text{O}}_{{{39}}}$$

**Figure 4:**
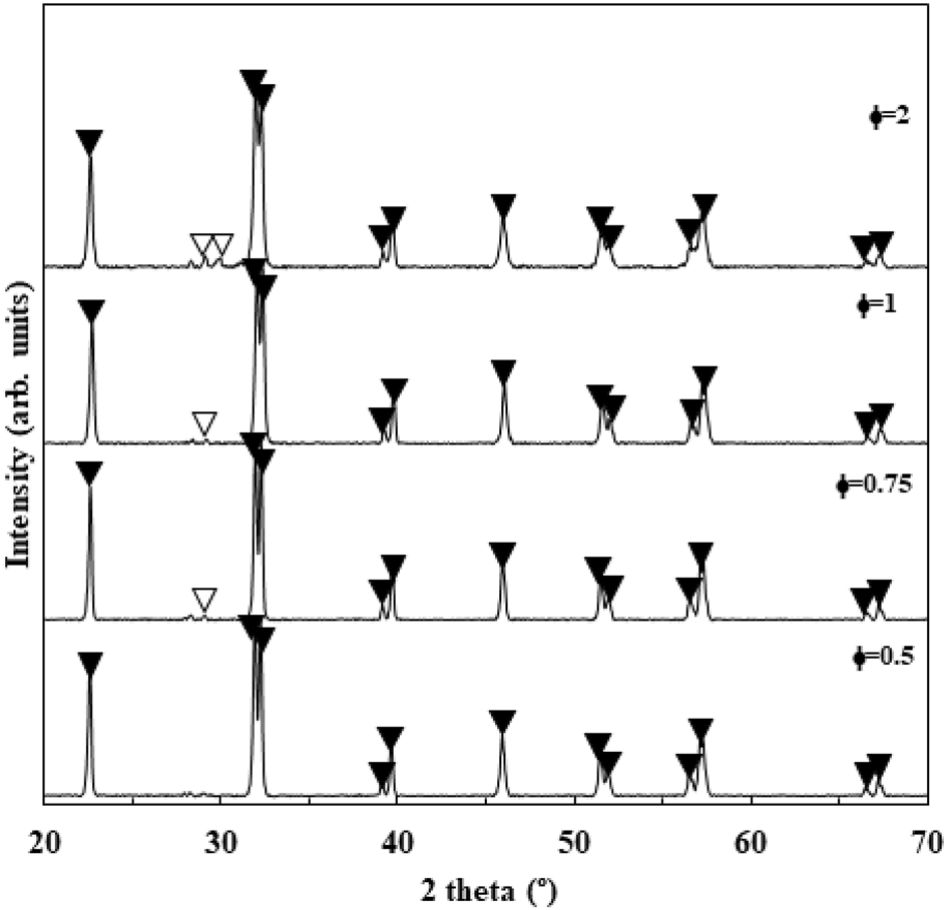
XRD patterns of the as-calcined BFO powders using glycine and CTAB fuel content (filled down pointing triangle: BiFeO_3_, open down pointing triangle: Bi_2_Fe_4_O_9_).

The inferior crystallinity of as-combusted powders due to their lower combustion temperatures can be improved by further calcination at higher temperatures (Fig. 4). Impurity phases were mainly eliminated by one-hour calcination at 600 °C due to the reaction of the residual Bi_2_O_3_ and Fe_2_O_3_ phases.

$${\text{Bi}}_{{2}} {\text{O}}_{{3}} + {\text{ Bi}}_{{2}} {\text{Fe}}_{{4}} {\text{O}}_{{9}} \to {\text{ 4 BiFeO}}_{{3}}$$

$${\text{11 Fe}}_{{2}} {\text{O}}_{{3}} + {\text{ Bi}}_{{{24}}} {\text{Fe}}_{{2}} {\text{O}}_{{{39}}} \to {\text{ 24BiFeO}}_{{3}}$$

**Figure 5:**
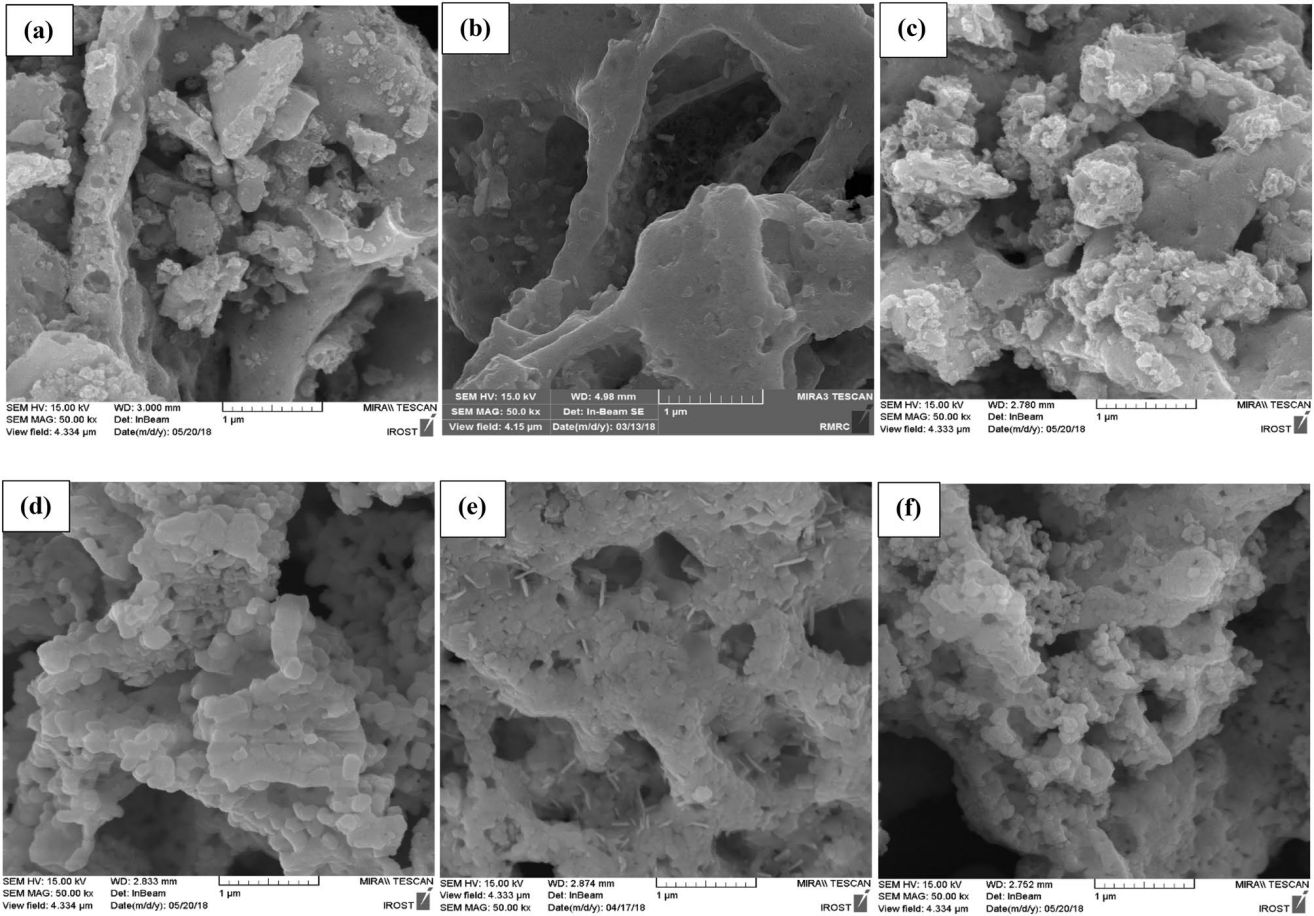
SEM micrographs of the as-combusted BFO powders at (**a**) φ = 0.75, (**b**) φ = 1 and (**c**) φ = 2 and the as-calcined BFO powders at (**d**) φ =0.75, (**e**) φ =1 and (**f**) φ =2.

SEM micrographs of BFO powders synthesized at different φ values are illustrated in Fig. 5. As combusted powders display a bulky microstructure, the particle sizes are reduced from 37 to 18 nm with the increase in fuel content presented in Table 1, as calculated from XRD data using the Williamson-Hall technique. Particle size mainly depends on the combustion temperature and reaction rate, where the combustion rate would influence the number of nucleation sites and higher combustion temperature enhances the particle growth^45^. When the fuel content is increased, a higher amount of generated heat is consumed by the combustion gases and hence reduces the adiabatic temperature. This decline in the adiabatic temperature resulted in particle size refinement. However, as seen in Fig. 5d–f, calcined powders exhibit particle size growth as a result of the rise in the temperature.

**Table 1:** Crystallite size (D_XRD_) of BiFeO_3_ phase, and bandgap energy (Eg) as a function of fuel type.

As-combusted	D_XRD_ (nm)	Eg (eV)
0.5	37	1.96
0.75	28	1.85
1	21	1.96
2	18	1.96

**Figure 6:**
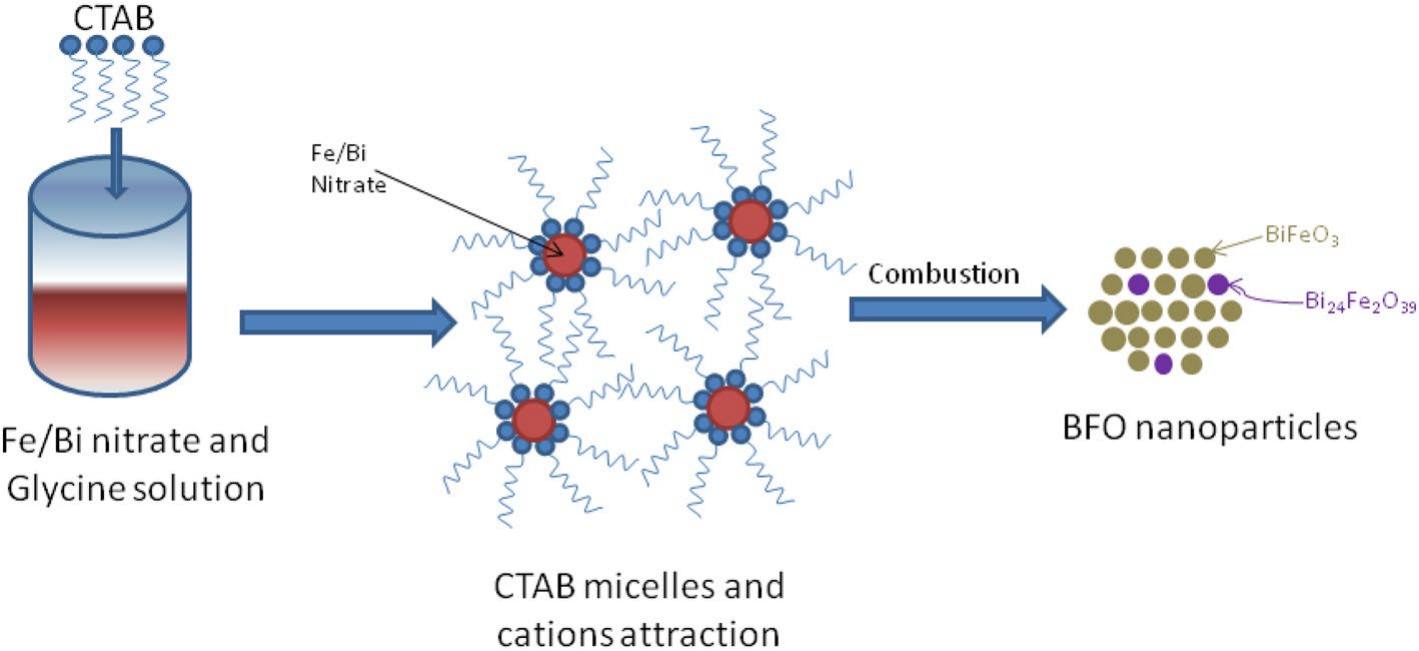
Schematic of BFO nanoparticle synthesis via micells formation at high CTAB content.

**Figure 7:**
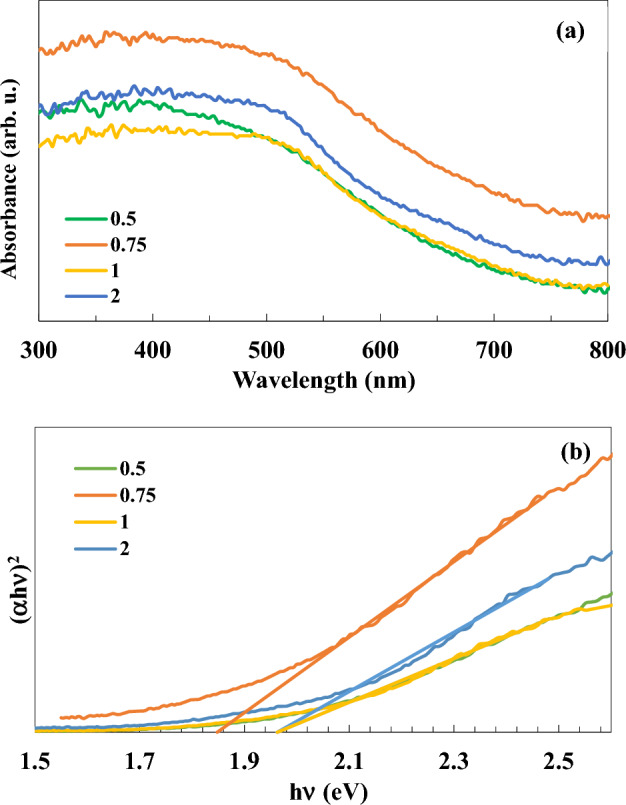
(**a**) UV–Vis diffuse reflectance spectra and (**b**) Tauc’s plot of the as-combusted BFO powders.

Another proposed reason behind this particle size refinement at higher CTAB/glycine content, as illustrated schematically in Fig. 6, could be due to the interaction of CTAB micelles with the cationic head inside the solution precursor, separated a large amount of the cationic ends apart and produced smaller BFO nanoparticles when the CTAB amount was maximized. Figure 7a displays diffuse reflectance spectra of the as combusted BFO powders. The amount of visible light absorption mainly depends on the crystallinity, strain, particle size, impurity phases, etc. The crystal field and metal–metal transitions affect the absorption spectra^46^. The increase in light absorption at higher fuel content (φ = 2) is possibly due to the decrease in the impurity phase Bi_24_Fe_2_O_39_. However, powders synthesized at fuel content of (φ = 0.75) significantly absorbed a higher amount of visible light, probably due to the formation of distinct impurity phase of Bi_2_Fe_4_O_9_, as previously discussed in XRD data. The Tauc’s plot measured bandgap energy of the combusted powders ((αhυ)^2^ vs. hυ), as shown in Fig. 7b and summarized in Table 1. The bandgap energies of the combusted BFO powders are in the range of 1.85–1.96 eV, in good agreement with the bandgap of powders and thin films reported in the literature^46^. At higher fuel contents, the decrease in bandgap energy is mainly due to the particle size refinement, while the increase in the bandgap energy at φ = 2 could be owed to the presence of the impurity phase Bi_24_Fe_2_O_39_.

**Figure 8:**
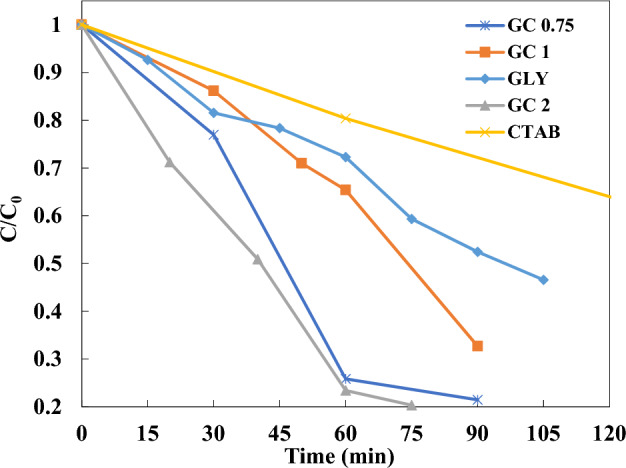
C/C_0_ versus irradiation time in the presence of the BFO powders at different fuel content and type.

The relative concentrations (C/C_0_) of MB, as an organic pollutant, versus visible light irradiation is illustrated in Fig. 8. The MB is photodegraded by about 80% during 90 min of visible light illumination for combusted powders at φ values of 2 and 0.75. However, the degradation rate is slightly higher for the combusted powders synthesized at φ = 2. Based on our previous findings^32,33^, the combusted powders synthesized by pure glycine or pure CTAB only showed the photodegradation of MB at about 50 and 30 percent, respectively. Therefore, by mixing the glycine and CTAB fuels, the photodegradation of MB is profoundly enhanced possibly due to the particle size refinement and higher crystallinity with less amount of impurity phases being present. The oxidation of MB dye molecule to CO_2_ and H_2_O species mainly depends on the presence of active species such as O^−2^, OH radicals^47^. The photogenerated electrons and holes during the light absorption of powders would react with the oxygen and water molecules to produce the active species. Thus, the optical properties of combusted powders such as bandgap energy, absorption coefficient, and band edge position play an important role in photocatalytic performance^48^.

## Conclusions

High purity BFO powders were synthesized via solution combustion synthesis by the mixture of glycine and CTAB as fuels at different fuel contents. The amount of the impurity phase was reduced by increasing fuel content from 0.5 to 2. Bi_2_Fe_4_O_9_ impurity phase was present at φ values of 0.5 and 0.75. However, at higher fuel ratios (φ values of 1 and 2), the impurity phase was transformed into the Bi_24_Fe_2_O_39_ phase.

The combusted powders at φ values of 2 and 0.75 showed the highest MB photodegradation of about 80% under 90 min of visible light illumination mainly due to the particle size refinement, higher visible light absorption, and less amount of impurities. Furthermore, the photodegradation rate of combusted powders synthesized at φ = 2 was somewhat enhanced.

## Data Availability

The datasets used and/or analysed during the current study available from the corresponding author on reasonable request.
